# Heart Rate Variability and Hemodynamic Change in the Superior Mesenteric Artery by Acupuncture Stimulation of Lower Limb Points: A Randomized Crossover Trial

**DOI:** 10.1155/2013/315982

**Published:** 2013-11-20

**Authors:** Soichiro Kaneko, Masashi Watanabe, Shin Takayama, Takehiro Numata, Takashi Seki, Junichi Tanaka, Seiki Kanemura, Yutaka Kagaya, Tadashi Ishii, Yoshitaka Kimura, Nobuo Yaegashi

**Affiliations:** ^1^Department of Obstetrics and Gynecology, Tohoku University Graduate School of Medicine, Japan; ^2^Comprehensive Education Center for Community Medicine, Tohoku University Graduate School of Medicine, 2-1 Seiryo-machi, Aoba Ward, Sendai-shi 980-8573, Japan; ^3^Department of Geriatric Behavioral Neurology, Tohoku University Graduate School of Medicine, Japan; ^4^Department of Education and Support for Community Medicine, Tohoku University Hospital, Japan

## Abstract

*Objective*. We investigated the relationship between superior mesenteric artery blood flow volume (SMA BFV) and autonomic nerve activity in acupuncture stimulation of lower limb points through heart rate variability (HRV) evaluations. *Methods*. Twenty-six healthy volunteers underwent crossover applications of bilateral manual acupuncture stimulation at ST36 or LR3 or no stimulation. Heart rate, blood pressure, cardiac index, systemic vascular resistance index, SMA BFV, and HRV at rest and 30 min after the intervention were analyzed. *Results*. SMA BFV showed a significant increase after ST36 stimulation (0% to 14.1% ± 23.4%, *P* = 0.007); very low frequency (VLF), high frequency (HF), low frequency (LF), and LF/HF were significantly greater than those at rest (0% to 479.4% ± 1185.6%, *P* = 0.045; 0% to 78.9% ± 197.6%, *P* = 0.048; 0% to 123.9% ± 217.1%, *P* = 0.006; 0% to 71.5% ± 171.1%, *P* = 0.039). Changes in HF and LF also differed significantly from those resulting from LR3 stimulation (HF: 78.9% ± 197.6% versus −18.2% ± 35.8%, *P* = 0.015; LF: 123.9% ± 217.1% versus 10.6% ± 70.6%, *P* = 0.013). *Conclusion*. Increased vagus nerve activity after ST36 stimulation resulted in increased SMA BFV. This partly explains the mechanism of acupuncture-induced BFV changes.

## 1. Introduction

In traditional medicine in East Asia, acupuncture therapy is achieved through acupoints, which are reactive points on the surface of the body. Each acupoint is thought to achieve specific and sometimes unique effects on human organ systems [1]. However, unique organ-specific effects associated with acupoints have been difficult to evaluate quantitatively, because of the lack of a quantitative method of evaluation of the effects of acupuncture. With this in mind, we have evaluated the effects on blood flow in the superior mesenteric artery, radial artery, brachial artery, and retrobulbar artery as a result of acupuncture stimulation at specific acupoints [2–6].

Acupuncture stimulation of ST36 is considered to have beneficial effects on gastrointestinal symptoms and has been used since ancient times [1]. We have reported that acupuncture stimulation of ST36 significantly increased superior mesenteric artery blood flow volume (SMA BFV) using ultrasonographic diagnostic equipment [5]. The mechanism of the increase in blood flow volume has not yet been fully elucidated but is speculated to result from vasodilation caused by the suppression of abdominal sympathetic nerve activity, promotion of abdominal vagus nerve activity, and increases in secondary blood flow due to promoted intestinal movement, as well as other mechanisms. 

Noninvasive methods are preferable to elucidate mechanisms by which the autonomic nervous system controls physiological phenomena such as increases in blood flow volume. In research and clinical practice, the spectral analysis of heart rate variability (HRV) has gained popularity and has gained wide popularity as a noninvasive monitor of the autonomic response [7]. Spectral analysis of HRV allows a quantitative evaluation of autonomic nerve activity by calculating the power of each frequency domain through a fast Fourier analysis of HRV obtained from the R-R interval on an electrocardiogram [7]. 

In the present study, we evaluated the involvement of autonomic nerve activity and changes in SMA BFV resulting from acupuncture stimulation of acupoints using a spectral analysis of HRV.

## 2. Method

**Figure 1:**
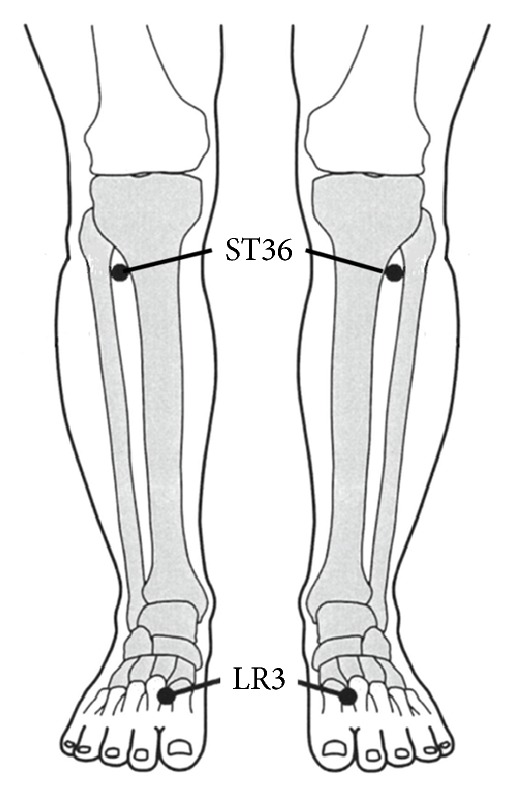
Locations of ST36 and LR3 where acupuncture stimulations were performed.

Crossover trials of stimulation at ST36 and LR3 and nonstimulation (CTL) were conducted (Figure 1). The subjects were 26 healthy volunteers (12 men and 14 women with a mean age of 28.9 ± 7.0 years). The subjects were randomly divided into 3 groups in which acupuncture stimulation of ST36, the same stimulation of LR3, and nonstimulation were conducted in a crossover fashion with at least 7 days between trials. 

Before each trial, resting measurements were obtained for each subject after 10 min of rest in the supine position at a room temperature of 25-26°C. Thereafter, acupuncture stimulation was performed by bilateral insertion of a number 1 needle (diameter: 0.16 mm, length: 40 mm; Seirin Co. Ltd., Shizuoka, Japan) at ST36 or LR3 to a depth of 1 cm over 18 sec, twirling the needle for 18 sec, and leaving the needle for 15 min. In the CTL group, the subject was observed for the same duration without acupuncture stimulation. An acupuncturist with over 5 years of acupuncture experience administered the acupuncture stimulation. ST36 is located on the lower leg, 3 units below the lateral “eye” of the knee and approximately 1 finger width lateral to the tibia [8]. LR3 is located on the foot, 1.5–2 units above the web between the first and second toes [8]. 

In each trial, heart rate (HR), systolic blood pressure (SBP), diastolic blood pressure (DBP), cardiac index (CI), systemic vascular resistance index (SVRI), SMA BFV, and power of each frequency domain calculated from HRV (very low frequency (VLF), low frequency (LF), high frequency (HF), and LF/HF) at rest (before) and 30 min after stimulation were extracted for analysis. The details of the extracted items were as follows.

### 2.1. HR and Spectral Analysis of HRV

**Figure 2:**
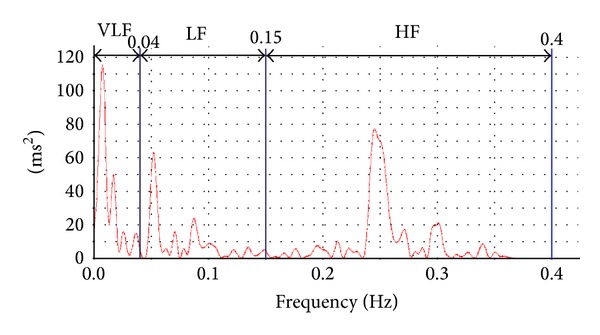
A spectrum screen in LabChart in which the power of each frequency domain (ms^2^) is calculated. The very low frequency (VLF), low frequency (LF), and high frequency (HF) domains are indicated in the figure. The solid red line in the figure represents the power calculated in each frequency band by spectral analysis.

In each trial, an electrocardiogram recorded values from rest until 30 min after acupoint stimulation. HR and HRV were recorded from electrocardiographic data obtained from a standard limb lead using the AD conversion system PowerLab (AD Instruments Pty Ltd., Australia). The data were extracted from the record for 3 min at rest and at 30 min after the intervention to avoid the period of the SMA BFV measurement using ultrasonographic diagnostic equipment. The data were subjected to a spectral analysis using LabChart analysis software (AD Instruments Pty Ltd., Australia) (Figure 2). The Task Force of the European Society of Cardiology and the North American Society of Pacing and Electrophysiology define 2–5 min as the extraction time required for a spectral analysis of HRV [7]. In the present experiment, to avoid the period of SMA BFV measurement required for the control of respiration, the extraction time for each period was set to 3 min. The sampling rate was 2 k/s.The baseline fluctuations were removed by a differential calculus wave pattern. The frequency domain analyses were performed using a nonparametric fast Fourier transform (FFT). The FFT spectra were then calculated using a Hann periodogram method and were as follows [7].

- VLF: very low frequency domain (≤0.04 Hz) power (ms^2^) generally reflects functions such as thermoregulation and renin-angiotensin system activity.
- LF: low frequency domain (0.04–0.15 Hz) power (ms^2^) generally reflects both the sympathetic and parasympathetic nervous systems.
- HF: high frequency domain (0.15–0.40 Hz) power (ms^2^) generally reflects the parasympathetic nervous system.
- LF/HF: the power ratio of LF to HF generally reflects the balance between the sympathetic and parasympathetic nervous systems.

### 2.2. SMA BFV

SMA BFV was measured within 2-3 cm from the origin of the artery using ultrasonographic diagnostic equipment (Prosound*α*10; Hitachi Aloka Co. Ltd., Tokyo, Japan). 

### 2.3. Blood Pressure Measurements (SBP and DBP)

SBP and DBP were measured using an oscillometer, BP-608 Evolution II (Colin Healthcare Co. Ltd., Tokyo, Japan).

### 2.4. CI

CI was calculated from intrathoracic impedance measured using 4 dual sensors of a BioZ ICG Module, Dash 3000 (GE Healthcare, USA).

For the statistical analysis, values of HR, SBP, CI, SVRI, SMA BFV, and the power of each HRV frequency domain (VLF, LF, HF, and LF/HF) at rest and 30 min after stimulation in each group were represented by the percentage change (%) relative to the resting value. The values before and after the intervention were compared by a paired *t*-test. A 2-sample *t*-test was used for intergroup comparisons. The difference was considered significant when the *P* value was less than 0.05. 

This study was conducted after approval by the Ethics Committee of Tohoku University School of Medicine. All subjects provided written consent regarding the contents of the experiments. 

## 3. Results

Tables 1–3 show the changes in the values 30 min after acupuncture stimulation at ST36 and LR3 and nonstimulation (CTL) compared to values before the intervention.  Table 4 shows a summary of changes in parameters.

In stimulation at ST36, VLF, LF, HF, and LF/HF significantly increased (*P* = 0.045, *P* = 0.006, *P* = 0.048, and *P* = 0.039, resp.), SBP significantly decreased (*P* = 0.037), and SMA BFV significantly increased (*P* = 0.007) (Table 1).

In LR3 stimulation, HF significantly decreased (*P* = 0.014) and LF/HF significantly increased (*P* = 0.013), while SMA BFV showed no significant change (Table 2).

In the CTL group, VLF and SVRI significantly increased (*P* = 0.047, *P* = 0.002) and CI significantly decreased (*P* < 0.001), while SMA BFV showed no significant change (Table 3).

In the comparison between ST36 and LR3 stimulations, a significant difference (*P* = 0.009) was observed in the change in SMA BFV as well as in LF and HF (*P* = 0.013, *P* = 0.015) (Table 5).

## 4. Discussion

In the present study, acupuncture stimulation at ST36 resulted in an increased SMA BFV together with significantly increased VLF, LF, HF, LF/HF, and significantly decreased SBP values. The intergroup comparison between the 2 acupoint stimulations showed significant differences in LF and HF.

### 4.1. Change in Parameters after ST36 Stimulation

The SMA supplies blood widely to the small and large intestines, and its blood flow is regulated by the peritoneal vagus and abdominal sympathetic nerves [9]. HF in HRV is considered to reflect parasympathetic nerve (cardiac vagus nerve) activity [7]. LF is considered to reflect both cardiac sympathetic nerve and cardiac vagus nerve activity [10]. LF/HF is considered to reflect the sympathovagal balance (balance between the sympathetic and parasympathetic nervous systems) [11, 12]. In the present experiment, ST36 stimulation significantly increased VLF, LF, HF, and LF/HF. Because CI, HR, and SVRI did not change while SBP decreased, we considered that the increase in SMA BFV induced by ST36 stimulation might have been caused by the augmentation of parasympathetic nerve (vagus nerve) activity rather than the suppression of sympathetic nerve activity. 

### 4.2. Changes in Parameters after LR3 Stimulation

LR3 stimulation caused no significant change in SMA BFV. In addition, when HR, SBP, CI, and SVRI showed no significant change, HF significantly decreased, LF/HF significantly increased, and LF showed no significant change. LR3 stimulation might have suppressed parasympathetic nerve (cardiac vagus nerve) activity while exerting no influence on sympathetic nerve activity.

### 4.3. Changes in Parameters in CTL

In CTL, while no significant change was seen in SMA BFV, CI significantly decreased, SVRI significantly increased, and HR and SBP showed no significant changes. While VLF significantly increased, HF, LF, and LF/HF showed no significant changes. Although there is a report stating that an increase of VLF indicates an enhancement of parasympathetic nerve activity [13], the increase of VLF was unlikely solely the result of an increase of parasympathetic nerve activity, because no change in HF, an indicator of parasympathetic nerve activity was seen. Therefore, it is likely that no changes in autonomic nerve activity related to HRV occur while resting in the supine position. 

### 4.4. Difference in Reaction Depending on the Acupoint Stimulated

In the comparison between ST36 and LR3 stimulations in the present experimental system, SMA BFV as well as HF and LF in HRV showed significant differences. These 2 acupoints are present in the same segment, that is, on the same afferent fiber. Mori et al. performed acupuncture stimulation at 2 acupoints in the same segment (afferent fiber) and indicated that the reaction of the autonomic nervous system may vary because of differences in the muscle (muscular segment), even though the input is made on the same afferent fiber [14]. Zhao et al. also mentioned the presence of acupoint specificity [15]. The presence of differences in SMA BFV and cardiac vagus nerve activity depending on the site (acupoint) in the present experimental system indicates that the reaction of the autonomic nervous system varies because of the difference in the site (acupoint), even though the acupuncture stimulation is applied to the same limb; this difference may be demonstrated by a spectral analysis of HRV. 

### 4.5. Interpretation of the Hypothesis in a Previous Study

Watanabe et al. speculated that increased SMA BFV after ST36 stimulation resulted from the suppression of the sympathetic nervous system through stimulation of the parasympathetic nervous system and spinal reflex [5]. In the present experiment, when ST36 stimulation caused an increase of the SMA BFV, we obtained a result indicating that activation of the parasympathetic nervous system increases in VLF, HF, LF, and LF/HF in HRV. This result supported the promotion of the parasympathetic nervous system, which was part of the hypothesis proposed by Watanabe et al.

## 5. Limitations

### 5.1. Effect of Respiratory Rate

HR effects due to sympathetic nerve activity have distinctly different characteristics from those due to vagus nerve activity. HRV does not exceed 0.15 Hz with sympathetic nerve transmission, whereas HRV up to around 1 Hz occurs with cardiac vagus nerve transmission because of differences in intracellular communication mechanisms downstream of *β* and Ach receptors [16]. To evaluate the HR effect of the cardiac vagus nerve separately from that of the sympathetic nerve, the frequency of the HF component needs to be maintained above 0.15 Hz (a respiratory rate of over 9 times/min), which is the limit of the frequencies that the sympathetic nerve can transmit [17, 18]. In the present study, the measurement items did not include respiratory rate. However, we speculate that the participants were breathing more than 9 times per min because we instructed them to stay awake during the trial, and healthy adults breathe 12–15 times per min at rest [19]. Further, a peak of more than 0.15 Hz is shown in the spectrum analysis in Figure 2. This peak is regarded to represent respiratory sinus arrhythmia, which confirms that the respiratory rate was more than 9 times/min.

### 5.2. Extraction Time in HRV Analysis

The Task Force of the European Society of Cardiology and the North American Society of Pacing and Electrophysiology define 2–5 min as the extraction time required for a spectral analysis of HRV [7]. Notably, VLF assessed from short-term recordings (i.e., <5 min) is considered unreliable and should be avoided. In the present experiment, to avoid the period of SMA BFV measurement required for the control of respiration, the extraction time was set to 3 min. To verify whether an accurate analysis could be conducted with extraction time of 3 min, we preliminarily compared VLF data from 5 min extractions with those from 3 min extractions. As a result, no significant difference was observed between the values of HRV calculated from 5 min and 3 min of data. The range of VLF components is less than 0.04 Hz. In other words, there are approximately 7 waves in 3 min and approximately 12 waves in 5 min in the VLF components. With the above analysis, we confirmed that there was no difference in data obtained from 7 and 12 waves. Therefore, we considered that the analytical results in the present experiment were valid, even though the extraction time was 3 min. 

### 5.3. Respiratory Control in the Measurement of SMA BFV

Because ultrasonographic diagnostic equipment is used to measure SMA BFV, respiration needs to be controlled for a short time during the measurement. We preliminarily investigated the presence or absence of the influence of respiratory control on HRV. We investigated the change in HRV after ST36 stimulation in a protocol without SMA BFV measurement (respiratory control) and compared it with that with SMA BFV measurement. As a result, no influence of respiratory control on HRV was seen. Thus, when examining multiple organs, we need to preliminarily check possible interference among examination procedures because multiple examinations need to be conducted simultaneously.

## 6. Conclusion

When SMA BFV increased after ST36 stimulation, HF and LF in HRV showed a significant increase and differed from those observed after LR3 stimulation. This indicated that an increase of vagus nerve activity most likely is involved in an increase of SMA BFV induced by ST36 stimulation. This result also demonstrated acupoint specificity.

## Figures and Tables

**Table 1:** Changes in parameters after acupuncture stimulation at ST36.

	Before	30 minutes after ST36 stimulation percentage change (%)	*P* value
VLF	0	479.4 ± 1185.6	0.045*
LF		123.9 ± 217.2	0.006*
HF		78.9 ± 197.6	0.048*
LF/HF		71.5 ± 171.1	0.039*
SBP		−2.6 ± 5.8	0.037*
DBP		−2.2 ± 9.0	0.252
HR		−0.4 ± 6.8	0.774
CI		−2.4 ± 5.7	0.053
SVRI		0.5 ± 9.6	0.791
SMA BFV		14.1 ± 23.4	0.007*

The values indicate the percentage change (%) calculated by setting the values at rest to 0 (%) and are represented by the mean ± standard deviation. *P < 0.05.

**Table 2:** Changes in parameters after acupuncture stimulation at LR3.

	Before	30 minutes after LR3 stimulation percentage change (%)	*P* value
VLF	0	90.0 ± 307.1	0.140
LF		10.6 ± 70.6	0.444
HF		−18.2 ± 35.8	0.014*
LF/HF		58.6 ± 113.5	0.013*
SBP		−0.3 ± 6.2	0.839
DBP		−0.8 ± 8.1	0.623
HR		−1.3 ± 7.8	0.432
CI		−1.4 ± 7.3	0.345
SVRI		1.2 ± 8.3	0.492
SMA BFV		−7.6 ± 31.4	0.250

The values indicate the percentage change (%) calculated by setting the values at rest to 0 (%) and are represented by the mean ± standard deviation. *P < 0.05.

**Table 3:** Changes in parameters after nonstimulation (CTL).

	Before	30 min after precondition percentage change (%)	*P* value
VLF	0	37.6 ± 93.8	0.047*
LF		60.2 ± 233.1	0.191
HF		69.6 ± 283.3	0.213
LF/HF		24.0 ± 93.3	0.194
SBP		−0.5 ± 5.9	0.713
DBP		−1.2 ± 7.9	0.453
HR		−1.3 ± 9.0	0.471
CI		−6.9 ± 5.8	<0.001*
SVRI		6.2 ± 8.5	0.002*
SMA BFV		−2.8 ± 17.0	0.432

The values indicate the percentage change (%) calculated by setting the values at rest to 0 (%) and are represented by the mean ± standard deviation. *P < 0.05.

**Table 4:** Summary of changes in parameters after ST36 stimulation, LR3 stimulation, and nonstimulation (CTL).

	30 minutes after stimulation or precondition		
	ST36	LR3	CTL
VLF	↑↑	→	↑↑
LF	↑↑	→	→
HF	↑↑	↓↓	→
LF/HF	↑↑	↑↑	→
HR	→	→	→
SBP	↓↓	→	→
DBP	→	→	→
CI	↓	→	↓↓
SVRI	→	→	↑↑
SMA BFV	↑↑	→	→

→: no change, ↑ or ↓: a trend (0.05 < *P* ≤ 0.1), and ↑↑ or ↓↓: a significant difference.

**Table 5:** Intergroup comparisons between ST36 and LR3 stimulations and between ST36 or LR3 stimulation and nonstimulation (CTL).

	ST36 versus LR3 (*P* value)	ST36 versus CTL (*P* value)	LR3 versus CTL (*P* value)
VLF	0.104	0.059	0.401
LF	0.013*	0.303	0.294
HF	0.015*	0.886	0.116
LF/HF	0.746	0.211	0.226
SBP	0.180	0.207	0.913
DBP	0.589	0.706	0.859
HR	0.683	0.686	0.978
CI	0.605	0.012*	0.007*
SVRI	0.806	0.041*	0.047*
SMA BFV	0.009*	0.006*	0.513

A 2-sample *t*-test was used for comparisons. *P < 0.05.
